# Lactoferrin and its digestive peptides induce interferon-α production and activate plasmacytoid dendritic cells ex vivo

**DOI:** 10.1007/s10534-022-00436-y

**Published:** 2022-08-26

**Authors:** Shutaro Kubo, Momoko Miyakawa, Asuka Tada, Hirotsugu Oda, Hideki Motobayashi, Sadahiro Iwabuchi, Shinobu Tamura, Miyuki Tanaka, Shinichi Hashimoto

**Affiliations:** 1 Food Ingredients and Technology Institute, R&D Division, Morinaga Milk Industry Co., Ltd., 1-83, 5, Higashihara, Zama, Kanagawa Japan; 2 Second Department of Surgery, Wakayama Medical University, 811-1 Kimiidera, Wakayama, Wakayama Japan; 3 Department of Molecular Pathophysiology, Wakayama Medical University, 811-1 Kimiidera, Wakayama, Wakayama Japan; 4 Department of Hematology/Oncology, Wakayama Medical University, 811-1 Kimiidera, Wakayama, Wakayama Japan

**Keywords:** Lactoferrin, Lactoferricin, Plasmacytoid dendritic cells (pDCs), HLA-DR, CD86, Interferon (IFN)

## Abstract

Plasmacytoid dendritic cells (pDCs) recognise viral single-stranded RNA (ssRNA) or CpG DNA via Toll-like receptor (TLR)-7 and TLR9, and produce interferon (IFN)-α. Activated pDCs upregulate human leukocyte antigen (HLA)-DR and CD86 expression levels. Ingestion of bovine lactoferrin (LF) activates pDCs, but little is known about its effects. In this study, the effects of LF and its pepsin hydrolysate (LFH) on the production of IFN-α from peripheral blood mononuclear cells (PBMCs) and pDCs were examined. PBMCs were prepared from peripheral blood of healthy adults and incubated with LF, LFH, or lactoferricin (LFcin) in the absence or presence of ssRNA derived from human immunodeficiency virus. The concentration of IFN-α in the supernatant and the expression levels of IFN-α, HLA-DR, and CD86 in pDCs were quantified by enzyme-linked immunosorbent assay and flow cytometry. In the absence of ssRNA, the concentration of IFN-α was negligible and LF had no effect on it. In the presence of ssRNA, IFN-α was detected at a certain level, and LF and LFH significantly increased its concentration. The increase caused by LFH and LFcin were comparable. In addition, LF significantly upregulated the expression levels of IFN-α, HLA-DR, and CD86 in pDCs. LF and its digestive peptides induced IFN-α production and activated pDCs in the presence of ssRNA, suggesting that LF modulates the immune system by promoting pDC activation upon viral recognition.

**Supplementary Information:**

The online version contains supplementary material available at 10.1007/s10534-022-00436-y.

## Introduction

Dendritic cells (DCs) act as a bridge between innate and adaptive immunity. They recognise invading pathogens and activate immune cells by producing cytokines and antigen presentation. Plasmacytoid DCs (pDCs) strongly express the Toll-like receptor (TLR)-7 and TLR9. TLR7 recognises viral single-stranded RNA (ssRNA), while TLR9 recognises CpG DNA derived from bacteria or viruses. Once pDCs recognise the pathogen genome via TLR7 and TLR9, they produce massive type I interferons [(IFN)-α/β]. Type I IFNs subsequently upregulate hundreds of interferon-stimulated genes that play important roles in the host antiviral response and further activate myeloid DCs, natural killer (NK) cells, CD4+ and CD8+ T cells, and B cells. pDCs also express co-stimulatory molecules (CD86) and major histocompatibility complex class II molecules (human leukocyte anti-gen (HLA)-DR) and present pathogen antigens to native T cells (Lande and Gilliet 2010; Perng and Lenschow 2018). Therefore, pDCs act as leaders of immune cells and modulate the innate and adaptive immunity. In fact, pDC deficiency worsens the severity of the respiratory syncytial virus (Smit et al. 2006), herpesvirus (Takagi et al. 2011; Jamali et al. 2020), and rotavirus (Deal et al. 2013) infections in vivo. In humans, pDCs and IFN-α play important roles in suppressing the influenza virus (Ciancanelli et al. 2015; Solov’ev et al. 1969), herpesvirus (Keles et al. 2014), and rotavirus (Moon et al. 2012; Mangiarotti et al. 2007; Soloviov et al. 2020) infections. These facts suggest that maintaining pDC activity in a suitable state is important for suppressing viral infection in the respiratory tract, gastrointestinal tract, and mouth/lip.

Lactoferrin (LF) is an iron-binding glycoprotein found in body fluids, such as milk, tears, and saliva. Currently, LF isolated from bovine milk is used as a functional food ingredient (Tomita et al. 2009). Oral administration of LF induces IFN-α/β production in Peyer’s patches and activates the NK (Kuhara et al. 2006), CD4+ and CD8+ T, and B cells in vivo (Arciniega-Martínez et al. 2015). In addition, ingestion of LF suppresses the symptoms of influenza virus (Shin et al. 2005), rotavirus (Pérez-Cano et al. 2008), and herpes-virus infections in vivo (Wakabayashi et al. 2004). Consistent with in vivo studies, intake of LF induces the gene expression of type I IFNs and activates the NK and CD4+ and CD8+ T cells in humans (Alexander et al. 2014; Iigo et al. 2014; Mulder et al. 2008). In healthy people, LF intake suppresses subjective symptoms in the respiratory tract (Motoki et al. 2020; Miyakawa et al. 2021a), gastrointestinal tract (Motoki et al. 2020; Mizuki et al. 2020), and mouth/lip (Miyakawa et al. 2021a).

Recently, we confirmed that the intake of LF (200 mg/day) enhances IFN-α and CD86 expression levels of pDCs in the peripheral blood mononuclear cells (PBMCs) of healthy subjects (Miyakawa et al. [Bibr CR21][Bibr CR22]). These findings suggest that LF intake modulates the innate and adaptive immunity by activating pDCs, leading to the maintenance of systemic health. However, little is known about the effect of LF on pDCs. Part of ingested LF is hydrolysed by the gastric protease, pepsin (Troost et al. 2001), and a cationic peptide named lactoferricin (LFcin) is released (Kuwata et al. 1998). LFcin represents part of the biological activities of LF such as antimicrobial activities (Wakabayashi et al. 2003). Therefore, both LF and its digestive peptides have a possibility to be involved in the immunomodulatory effects of ingested LF.

In this study, we investigated the effects of LF, pepsin hydrolysate of LF (LFH), and synthesised LFcin on the activation of immune system on PBMCs and pDCs in healthy adult donors, stimulated with ssRNA derived from human immuno-deficiency virus.

## Materials and methods

### Preparation of test materials

LF isolated from skimmed milk with a purity of 97.2% and iron content of 17.8 mg/100 g was used in this study (Morinaga Milk Industry, Japan). The endotoxin content of LF was found to be 0.83 EU/mg, using the limulus amoebocyte lysate (LAL) assay kit (Seikagaku Corporation, Japan).

LFH was prepared as previously described (Tomita et al. 1991). LF was dissolved in distilled water (50 mg/mL), and the pH was adjusted to 3.0 with HCl. Porcine pepsin (10 U/mg) (Difco Laboratories, MI, USA) was added (1.5 mg/mL) and LF was hydrolysed at 37 °C for 4 h. The reaction was terminated by heating at 80 °C for 10 min. The pH was re-adjusted to 6.0 using NaOH, and the hydrolysate solution was lyophilised. The endotoxin content of LFH was found to be 0.22 EU/mg, using the LAL assay.

LFcin (residues 17–42 of LF), having an intramolecular disulphide bond, is a peptide contained in LFH (Wakabayashi et al. 2003). LFcin was synthesised using the 9-fluorenylmethoxy carbonyl (Fmoc) method at the Toray Research Center, Inc., Japan. Purity (> 95%) was confirmed using reverse-phase high-performance liquid chromatography.

As the molecular weight of LF (approximately 80,000) is about 25-fold that of LFcin (3,195), to compare in the same molecular concentration, LF and LFH were dissolved in distilled water at 10 mg/mL, and LFcin was at 0.4 mg/mL, and filter sterilised. Final concentrations of LF and LFH in this study were set to 100 μg/mL as 100 μg/mL of human LF were found to activate human DCs derived from monocytes in a previous study (Spadaro et al. 2008).

ssRNA40/LyoVec (Invitrogen, Carlsbad, CA, USA) is an ssRNA derived from the human immuno-deficiency virus-1. It induces IFN-α production by pDCs in PBMCs via TLR7 signalling (Zhou et al. 2012). It was aseptically dissolved in sterilised distilled water at a concentration of 25 µg/mL.

### Preparation of PBMCs

After written informed consent was obtained from 11 healthy Japanese adult donors in Wakayama Medical University (Table 1), peripheral blood was collected in a Vacutainer CPT tube (BD, USA). The tubes were centrifuged at 1,500×*g* at 25 °C for 20 min. The fraction containing the mononuclear cells was harvested and washed with cold phosphate-buffered saline (PBS) (Fujifilm Wako, Japan). Contaminated red blood cells were hemolysed using ammonium chloride solution (STEMCELL TECHNOLOGIES, Canada) at 25 °C for 10 min. After haemolysis, the cells were washed with cold PBS, counted, resuspended in the Roswell Park Memorial Institute-1640 medium (Sigma-Aldrich, USA) supplemented with 5% human AB serum (Sigma-Aldrich, USA), 100 U/mL of penicillin, and 100 μg/mL of streptomycin (Thermo Fisher Scientific, USA), and kept on ice until the start of the experiment (1 × 10^6^ cells/890 μL). Due to the limited number of obtained PBMCs, peripheral blood was obtained repeatedly from part of donors, and used for each experiment. As frozen PBMCs did not produce IFN-α, only fresh PBMCs were used in this study.

**Table 1:** Characteristics of PBMCs donors

Age (years)	Total	Men	Women
20–29	1	1	0
30–39	5	3	2
40–49	4	2	2
50–59	1	1	0
Total	11	7	4
Mean age (years)	39.1	37.7	41.5

### Labelling of dead PBMCs to evaluate their viabilities

In a 24-well plate, 445 µL of PBMCs (5 × 10^5^ cells), 5 μL of sterilised distilled water, LF, or LFH solution (final concentration of 100 μg/mL), and 50 μL of sterilised distilled water were added to each well to make a final volume of 500 µL (n = 3). They were incubated at 37 °C for 24 h in humidified 5% CO_2_ atmosphere. Cells were harvested, washed with cold PBS, and treated with Horizon Fixable Viability Stain (BD Biosciences, USA) at 25 °C for 15 min to stain the dead cells. Cells were washed with the Stain Buffer (foetal bovine serum) (BD Biosciences, USA) and treated with 4% paraformaldehyde for 20 min on ice. Fixed cells were washed with Stain Buffer, resuspended in 500 μL of Stain Buffer, and stored at 4 °C until measurement. Flow cytometry was performed, as described below. The viabilities of PBMCs were calculated using the following formula:

$${\text{PBMC viability }}\left( \% \right) \, = {\text{ number of live PBMCs}}/{\text{number of total PBMCs }} \times { 1}00$$

### Measurement of IFN-α released from PBMCs

Experiment 1: In a 24-well plate, 445 µL of PBMCs (5 × 10^5^ cells) and 5 μL of sterilised distilled water or LF solution (final concentration, 100 μg/mL) were added. Then, 50 μL of sterilized distilled water was added to each well instead of the ssRNA solution to a final volume of 500 µL (n = 4). The pH value of the control medium was 7.24 and that of LF-added medium was 7.21.

Experiment 2: In a 24-well plate, 445 µL of PBMCs (5 × 10^5^ cells), 5 μL of sterilised distilled water or LF solution (final concentration, 100 μg/mL), and 50 μL of ssRNA solution (final concentration, 2.5 µg/mL) were added (n = 6). The pH value of the ssRNA-added medium was 7.20 and that of ssRNA and LF-added medium was 7.26.

Experiment 3: In a 24-well plate, 445 µL of PBMCs (5 × 10^5^ cells), 5 μL of sterilised distilled water or LFH solution (final concentration, 100 μg/mL), and 50 μL of ssRNA solution (final concentration, 2.5 µg/mL) were added (n = 4). The pH value of the medium containing ssRNA and LFH was 7.18.

Experiment 4: In a 96-well plate, 178 µL of PBMCs (2 × 10^5^ cells), 2 μL of LFH solution (final concentration, 100 μg/mL) or LFcin solution (final concentration, 4 μg/mL) and 20 μL of ssRNA solution (final concentration, 2.5 µg/mL) were added (n = 5). The pH value of the medium containing ssRNA and LFcin was 7.18.

The ssRNA and LF, LFH, or LFcin solutions were added simultaneously. The plates were incubated at 37 °C for 24 h under humidified 5% CO_2_ conditions, harvested into tubes, and centrifuged at 500×*g* at 25 °C for 5 min to collect the supernatant. The concentration of IFN-α in the supernatant was quantified by using the enzyme-linked immunosorbent assay (ELISA) kit (PBL Assay Science, USA). The assay range for the ELISA kit was 1.95–125 pg/mL.

### Labelling the intracellular expression levels of IFN-α in pDCs

In a 24-well plate, 445 µL of PBMCs (5 × 10^5^ cells), 5 μL of sterilised distilled water or LF solution (final concentration, 100 μg/mL), and 50 μL of ssRNA solution (final concentration, 2.5 µg/mL) were added (n = 10). They were incubated at 37 °C for 20 h under humidified 5% CO_2_ conditions. At 8 h, 0.5 μL of protein transport inhibitor (BD Biosciences, USA) was added to the medium to accumulate IFN-α produced in the cells. At 20 h, the cells were harvested, washed with the Stain Buffer, and treated with Human BD Fc Block (BD, USA) for 10 min on ice to prevent non-specific binding of antibodies. Cells were then treated with fluorescent-conjugated antibodies, CD123-PE-Cy7 (7G3), CD304-BB515 (U21-1283), and HLA-DR-PE (G46-6) for 30 min under shading conditions on ice. Cells were washed with Stain Buffer, and fixed with 4% paraformaldehyde for 20 min on ice. Fixed cells were washed with Stain Buffer, resuspended, and kept in Stain Buffer overnight at 4 °C. The next day, the cells were treated with the Perm/Wash Buffer (BD Biosciences, USA) for 15 min on ice to permeabilise them. Permeabilised cells were treated with IFN-α-Alexa Fluor 647 (7N4-1) at 25 °C for 1 h under shading conditions to stain the intracellular IFN-α. The cells were washed with the Perm/Wash Buffer, resuspended in 500 μL of Perm/Wash Buffer, and stored at 4 °C until measurement.

### Labelling the cell surface CD86 and HLA-DR expression levels of pDCs

In a 24-well plate, 445 µL of PBMCs (5 × 10^5^ cells), 5 μL of sterilised distilled water or LF solution (final concentration, 100 μg/mL), and 50 μL of ssRNA solution (final concentration, 2.5 µg/mL) were added (n = 10). They were incubated at 37 °C for 24 h in humidified 5% CO_2_ atmosphere. Cells were harvested, washed with cold PBS, and treated with the Horizon Fixable Viability Stain at 25 °C for 15 min to stain the dead cells. The cells were then washed with Stain Buffer and treated with the Human BD Fc Block for 10 min on ice. Cells were then treated with fluorescent-conjugated antibodies [CD123-PE-Cy7 (7G3), CD304-BB515 (U21-1283), CD86-APC (2331), HLA-DR-PE (G46-6)] or their isotype controls (BD Biosciences, USA) for 30 min under shading conditions on ice. Cells were washed with Stain Buffer, and treated with 4% paraformaldehyde for 20 min on ice. Fixed cells were washed with Stain Buffer, resuspended in 500 μL of Stain Buffer, and stored at 4 °C until measurement.

### Flow cytometry

Labelled cells were analysed using Cyto Flex (Beckman Coulter, USA). Data analysis was performed using the CytExpert 2.4 software (Beckman, USA). The gating strategy used to observe pDCs in PBMCs was almost the same as that used in our previous study (Kubo et al. 2021). Briefly, the first set of total PBMCs was gated on a forward scatter (FSC)/side scatter (SSC) plot. Next, the live cells were gated on a fixable viability stain/SSC plot. Doublets were excluded from the FSC height (FSC-H)/FSC width (FSC-W) plot and single cells were selected. Subsequently, CD123 + CD304 + cells were defined as pDCs, and cell surface expression levels of HLA-DR and CD86 in pDCs were observed (Supplementary Fig. S1). Similarly, the first set of total PBMCs was gated on an FSC/SSC plot. Doublets were excluded from the FSC-H/FSC-W plot and single cells were selected. CD123 + CD304 + cells were defined as pDCs, and the intracellular expression levels of IFN-α in pDCs were observed.

### Statistical analyses

The viabilities of PBMCs were evaluated using the paired Student's t-test. IFN-α concentration in the supernatant was logarithmically transformed (Log10) to normalise the data. Paired Student's t-test was used for the comparison. The percentages of IFN-α-, CD86-, or HLA-DR-positive pDCs in total pDCs and the median fluorescence intensity (MFI) of each marker in pDCs were not normally distributed, as evaluated by the Wilcoxon Signed-rank Test. Statistical analyses were performed using the EZR software version 1.54 (Saitama Medical Center, Jichi Medical University, Saitama, Japan) (Kaneda 2013). Statistical significance was set at P < 0.05.

## Results

### Viability of PBMCs

The mean viability of PBMCs treated with LF (100 µg/mL) was 93.3% and that of the control PBMCs (treated with water) was 94.5%. The viability of PBMCs treated with LFH (100 µg/mL) was 96.5% and that of the control PBMCs (treated with water) was 96.0%. The viabilities treated with LF or LFH were not significantly different from those of the control (Supplementary Fig. S2).

### IFN-α production from PBMCs in absence of ssRNA

In the absence of ssRNA, IFN-α concentrations in the culture supernatants of PBMCs were below the lower limit of quantification. Treatment with LF (100 µg/mL) did not alter the IFN-α concentration. (n = 4) (Supplementary Fig. S3).

### IFN-α production from PBMCs in presence of ssRNA

**Fig. 1:**
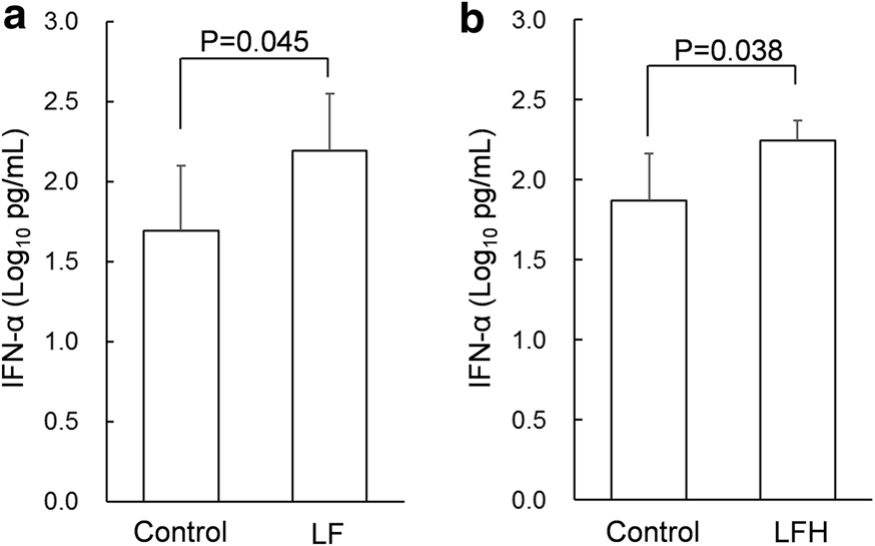
Concentrations of interferon (IFN)-α in the culture supernatants of peripheral blood mononuclear cells (PBMCs) treated with water (control) and 100 µg/mL of LF (**a**) (n = 6) or its pepsin hydrolysate (LFH) (**b**) (n = 4) in the presence of single-stranded RNA (ssRNA) for 24 h. The bars and error bars represent the mean and standard deviation

In the presence of ssRNA, IFN-α was detectable in the culture supernatants of PBMCs. Mean log transformed IFN-α concentration (Log10 pg/mL) in the culture supernatant of PBMCs treated with LF (100 µg/mL) was 2.19, while that in the control PBMCs (treated with water) was 1.69. Treatment with LF significantly enhanced the IFN-α concentration (Fig. 1a). Next, we evaluated IFN-α production in PBMCs treated with LFH. Mean log transformed IFN-α concentration in the culture supernatant of PBMCs treated with LFH (100 µg/mL) was 2.25, while that in the control PBMCs (treated with water) was 1.87. Although LFH lost the protein structure of LF, treatment with LFH unexpectedly enhanced the IFN-α concentration (Fig. 1b). In a supplementary experiment, PBMCs collected from one donor were treated with LF (100 µg/mL) or LFH (100 µg/mL), or water (control). As a result, LF and LFH seemed to enhance IFN-α production from PBMCs to same extent (Supplementary Fig. S4).

### A peptide promoting IFN-α production

**Fig. 2:**
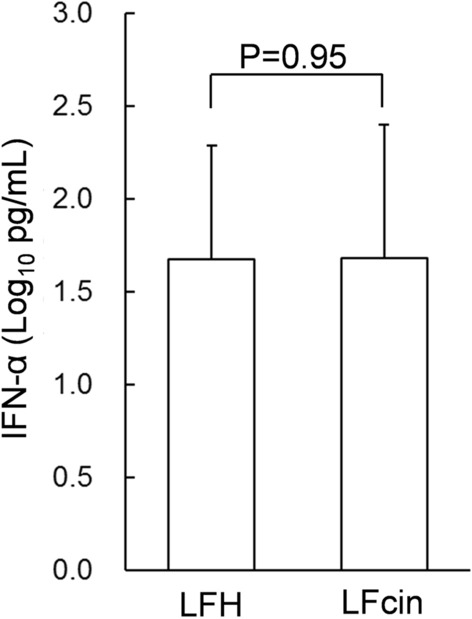
Concentrations of IFN-α in the culture supernatants of PBMCs treated with 100 µg/mL of LFH or 4 µg/mL of lactoferricin (LFcin) in the presence of ssRNA for 24 h. The bars and error bars represent the mean and standard deviation (n = 5)

LFH contains various peptides. These peptides were analyzed using HPLC in the previous study (Oda et al. 2013). LFcin was presented the largest peak of HPLC chromatogram analyzing LFH. In addition, LFcin is a characteristic peptide in its strong positive charge among LFH. Therefore, LFcin was expected to be one of the candidate peptides involved in promoting IFN-α production. To compare LFH and the same number of LFcin molecules included in LFH, PBMCs collected from one donor were treated with LFH (100 µg/mL), LFcin (4 µg/mL), or water (control) as a preliminary experiment, in the presence of ssRNA. As expected, LFcin seemed to increase IFN-α production equivalent to LFH compared with the control, and to be the major peptide promoting IFN-α production included in LFH (Supplementary Fig. S5). Considering the PBMC yield, PBMCs from five donors were treated with LFH and LFcin in the presence of ssRNA to evaluate whether LFcin enhanced IFN-α production from PBMCs similar to LFH. Means of the log transformed IFN-α concentration (Log10 pg/mL) in the culture of PBMCs treated with LFH (100 µg/mL) was 1.67 and that treated with LFcin (4 µg/mL) was 1.68. In the presence of ssRNA, the concentrations of IFN-α in the culture supernatants of PBMCs treated with LFH and LFcin were comparable, and there was no significant difference between them (Fig. 2).

### Intracellular IFN-α expression in pDCs in presence of ssRNA

**Fig. 3:**
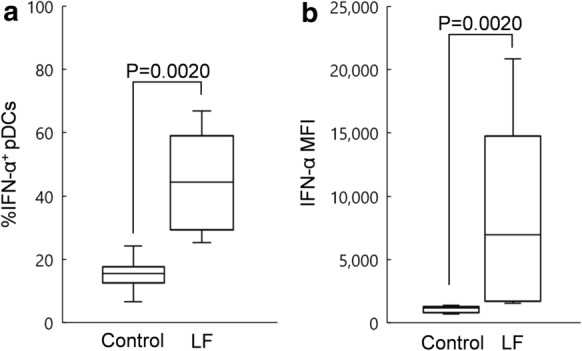
Percentage of IFN-α^+^ plasmacytoid dendritic cells (pDCs) in total pDCs (a) and the MFI of IFN-α in pDCs (b) treated with water (control) or 100 µg/mL of LF in the presence of ssRNA for 20 h. The horizontal line indicates the median, the box covers the 25–75th percentiles, and the vertical whiskers show the highest and lowest values excluding outliers (n = 10)

As LF, LFH, and LFcin similarly induced IFN-α production in PBMCs, we focused on the effect of LF on pDCs in PBMCs. In the presence of ssRNA, the median percentage of IFN-α^+^ pDCs among total pDCs present in PBMCs treated with LF (100 µg/mL) was 44.43%, while that in the control PBMCs (treated with water) was 15.59% (Fig. 3a). The median MFI of IFN-α in pDCs present in PBMCs treated with LF (100 µg/mL) was 6,949, while that in the control (treated with water) was 1,153 (Fig. 3b). In the presence of ssRNA, treatment with LF significantly increased the percentage of IFN-α^+^ pDCs in total pDCs and the MFI of IFN-α in pDCs.

### Cell surface expression of CD86 and HLA-DR in pDCs in presence of ssRNA

**Fig. 4:**
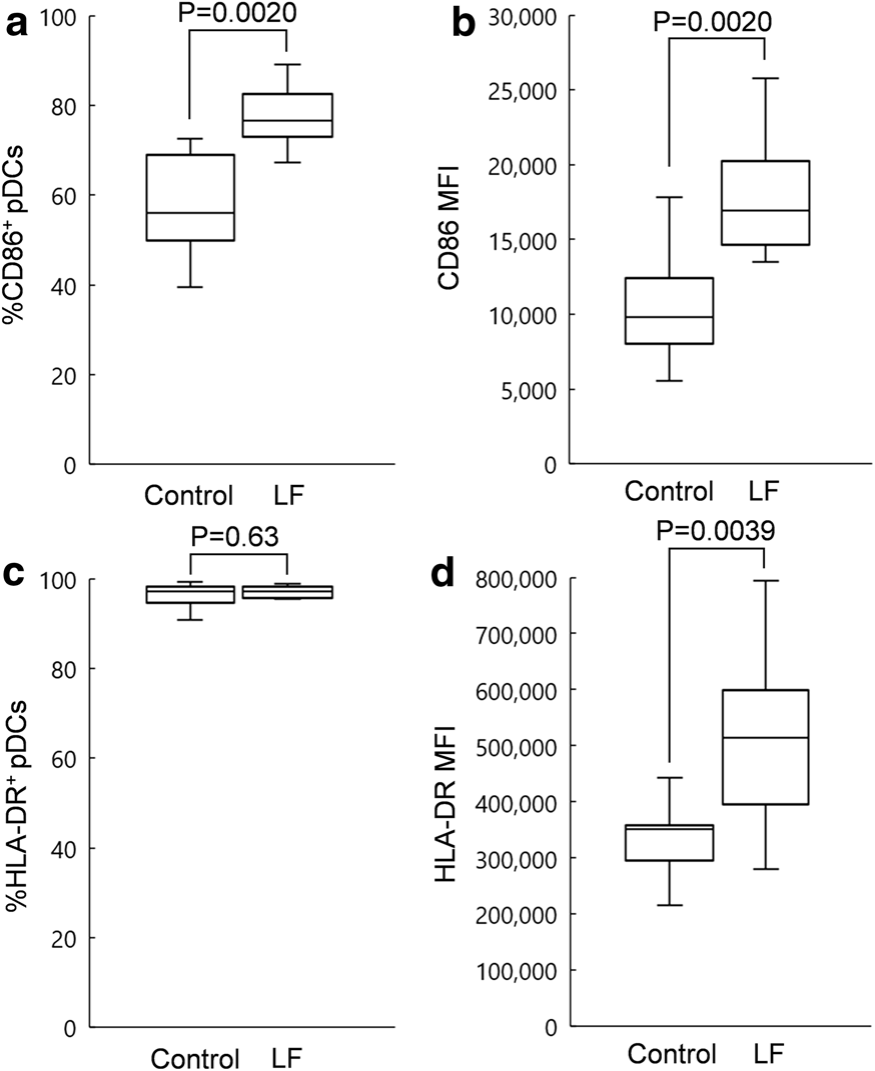
Percentage of CD86^+^ pDCs in total pDCs (**a**), MFI of CD86 in pDCs (**b**), percentage of human leukocyte antigen (HLA)-DR^+^ pDCs in total pDCs (**c**), and MFI of HLA-DR in pDCs (**d**) treated with water (control) or 100 µg/mL of LF in the presence of ssRNA for 24 h. The horizontal line indicates the median, the box covers the 25–75th percentiles, and the vertical whiskers show the highest and lowest values excluding outliers (n = 10)

In the presence of ssRNA, the median percentage of CD86^+^ pDCs, among total pDCs present in PBMCs treated with LF (100 µg/mL), was 76.56%, while that in control PBMCs (treated with water) was 55.98% (Fig. 4a). The median MFI of CD86 in pDCs treated with LF (100 µg/mL) was 16,912, while that in the control (treated with water) was 9,802 (Fig. 4b). In the presence of ssRNA, treatment with LF significantly increased the percentage of CD86^+^ pDCs among the total pDCs and the MFI of CD86 in pDCs. In the presence of ssRNA, the median percentage of HLA-DR^+^ pDCs, among total pDCs present in PBMCs treated with LF (100 µg/mL), was 97.12%, while that in the control PBMCs (treated with water) was 97.09% (Fig. 4c). The median MFI of HLA-DR in pDCs treated with LF (100 µg/mL) was 514,555, while that in the control (treated with water) was 350,775 (Fig. 4d). In the presence of ssRNA, treatment with LF did not change the percentage of HLA-DR + pDCs among the total pDCs but significantly increased MFI of CD86 in pDCs.

## Discussion

In this study, we examined whether LF and its digestive peptides induced IFN-α production in PBMCs. We found that LF and LFH did not affect the viabilities of PBMCs. PBMCs did not produce IFN-α in the absence of ssRNA. This is reasonable because the donors of peripheral blood were healthy and did not have any autoimmune diseases or infections. LF did not increase IFN-α production in the absence of ssRNA, which suggests that LF does not induce IFN-α production in the absence of viral RNA. In contrast, in the presence of ssRNA, PBMCs produced IFN-α, and both LF and LFH significantly enhanced IFN-α production. This is similar to our previous studies showing that LF induces the production of IFN-α/β/λ in intestinal epithelial cells in the presence of poly (I:C), a TLR3 agonist (Miyakawa et al. 2021a; Shin et al. 2017). Poly (I:C) is a double-stranded RNA that mimics viral replication intermediates. Therefore, LF may synergistically induce IFN production with viral RNA, probably owing to their cation–anion interactions. In contrast, LF inhibits the immune response induced by bacterial lipopolysaccharide (LPS), a TLR4 agonist (Drago-Serrano et al. 2012). The immunomodulatory effects of LF may differ in viral and bacterial infections. LFH is a mixture of various peptides derived from LF, and contains a distinctive cationic peptide with 26 amino acid residue, LFcin (Wakabayashi et al. 2003). LFcin has been widely investigated as an antimicrobial peptide; however, little is known about its immunomodulatory effects. We confirmed that LFH and LFcin, at the same molecular concentration, induced IFN-α production to the same degree. This suggests that LFcin is the active peptide in LFH that induces IFN-α production. This is the first study to suggest the involvement of LFcin in IFN-α production.

Commercial bovine LF contains a low percentage of LPS (Lönnerdal et al. 2021). LPS itself does not promote IFN-α production from pDCs but enhances IFN-α production in the presence of TLR-7/9 agonists, owing to cross-reactivity with TLR-4 pathways (Dai et al. 2004). However, in this study, the LPS content in LF (0.83 EU/mg) and LFH (0.22 EU/mg) was low enough to not cause any cross-reactivity. According to previous reports, to stimulate TLR-4 by LPS, more than about 10 ng/mL of LPS, which is approximately equivalent to 100 EU/mL, is needed. Therefore, the effects of endotoxin contained in the samples on this experimental system was assumed to be negligible.

In humans, more than 60% of LF administered to the stomach survives, and less than 40% is digested (Troost et al. 2001). In addition, LFcin is detected in the human stomach upon the intake of LF (Kuwata et al. 1998). Intelectin, a receptor for LF, is expressed on the epithelia of the small intestine, where both LF and LFH can bind (Shin et al. 2008). Therefore, the mixture of LF and its digestive peptides may interact with the small intestine. LF is a large molecule with a molecular weight of approximately 80,000 that is slightly absorbed via the lymphatic vessels (Takeuchi et al. 2004). LFcin and its further digestive peptides are smaller than LF; therefore, they may be absorbed more easily and act on immune cells in the peripheral blood. In the bovine intestine, LF binds to epithelia, especially that overlaying the Peyer’s patches (Talukder et al. 2003). In vitro microfold (M) cell model transcytosed LF from the apical to the basolateral side (Mulligan et al. 2006). Therefore, ingested LF and LFcin released in the stomach may act on immune cells in Peyer’s patches and induce IFN-α production, as observed in vivo (Kuhara et al. 2006). The digestive tract is continuously exposed to exogenous antigens, and the genomes of various viruses are detected in the lymphoid tissues of the digestive tract (Proenca-Modena et al. 2012). These viruses may be donors of ssRNA, and LF and LFcin may enhance the sensitivity of immune cells to ssRNA. However, because the characteristics of immune cells in the digestive tract are different from those in the peripheral blood (Vangeti et al. 2019), further studies using immune cells isolated from lymphoid tissues, such as tonsils and Peyer’s patches, are required to confirm our hypothesis.

As pDCs are the major producers of IFN-α in PBMCs, we examined whether LF activated pDCs in PBMCs in the presence of ssRNA. As expected, LF enhanced intracellular IFN-α, cell surface CD86, and HLA-DR expression levels in pDCs. In our preliminary study, LF enhanced cell surface CD86 expression levels, without changing HLA-DR expression levels in pDCs in the absence of ssRNA (Supplementary Fig. S6) (Kubo et al. 2021). These findings are in agreement with our previous trial results revealing that LF intake increased the intracellular IFN-α and cell surface CD86 expression levels in pDCs in PBMCs (Miyakawa et al. [Bibr CR21][Bibr CR22]). In another clinical trial, LF intake enhanced TLR7-mediated responses in pDCs in elderly women (van Splunter et al. 2018). Therefore, ingested LF may act on pDCs and facilitate IFN-α production and antigen presentation upon recognition of viral ssRNA. We could not evaluate the effects of LFH and LFcin on pDCs in PBMCs due to the limited number of PBMCs obtained for this study. We would like to examine this in the future.

In this study, we evaluated the effects of LF on pDCs in PBMCs, but not on isolated pDCs. Our results include the possibility LF affected pDCs via cooperation with other cells present in PBMCs. However, there are reports indicating that LF modulates antigen-presenting molecules such as CD86 and HLA-DR in macrophages (Hwang et al. 2009) or isolated bone marrow-derived DCs (Hwang and Actor 2009). These reports suggest that LF directly modulates the expression of antigen-presenting molecules on antigen-presenting cells (APCs) through a common mechanism. Therefore, it will be interesting to determine the mechanism by which LF modulates the expression of APCs, including pDCs. The mitogen-activated protein kinase (MAPK) signaling pathway, especially the p38 signaling process, modulates the expression of antigen-presenting molecules (Nakahara et al. 2006). Moreover, LF modulates the MAPK pathway in macrophages via TLR4-dependent and -independent signaling pathways (Curran et al. 2006). Therefore, LF may directly upregulate the expression of antigen-presenting molecules in pDCs by modulating the MAPK signaling pathway.

In summary, our findings suggest a novel mechanism by which LF modulates the immune system. Ingested LF has a possibility to act on pDCs, enhance IFN-α production and antigen presentation upon viral recognition, modulate the innate and adaptive immunity, and protect systemic health from viral infections.

## Conclusions

In this study, we found that LF and its digestive peptides induced IFN-α production and activated pDCs in the presence of ssRNA, suggesting that LF modulates the immune system by promoting pDC activation upon viral recognition.

## Supplementary Information

Below is the link to the electronic supplementary material.

Supplementary file1 (DOCX 771 KB)
