# Supplementary material for: Lactoferrin and its digestive peptides induce interferon-α production and activate plasmacytoid dendritic cells ex vivo
Source: Biometals. 2022 Aug 26;36(3):563–73. doi: 10.1007/s10534-022-00436-y (PMC10181974; doi:10.1007/s10534-022-00436-y)
Supplement: Supplementary file 1 — Supplementary file1 (DOCX 771 KB) [file 10534_2022_436_MOESM1_ESM.docx]

Supplementary data for Lactoferrin and its digestive peptides induce interferon-α production

and activate plasmacytoid dendritic cells *ex vivo*


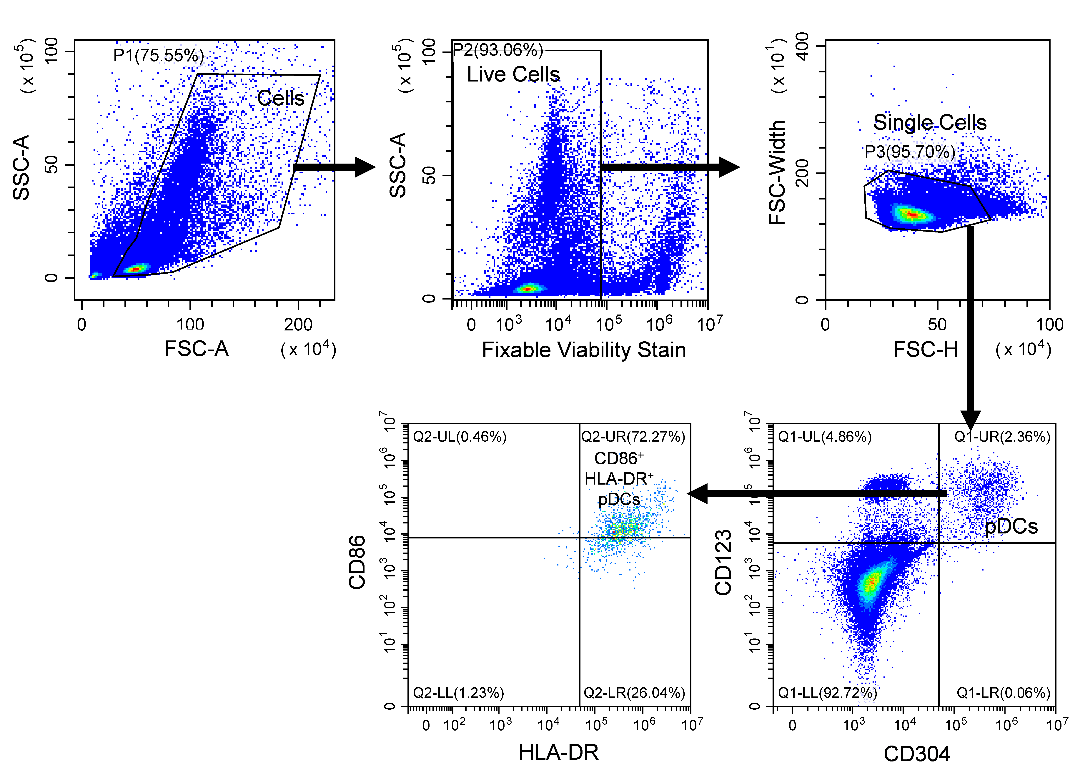


**Supplementary Fig. S1** Gating strategy to observe pDCs in PBMCs.


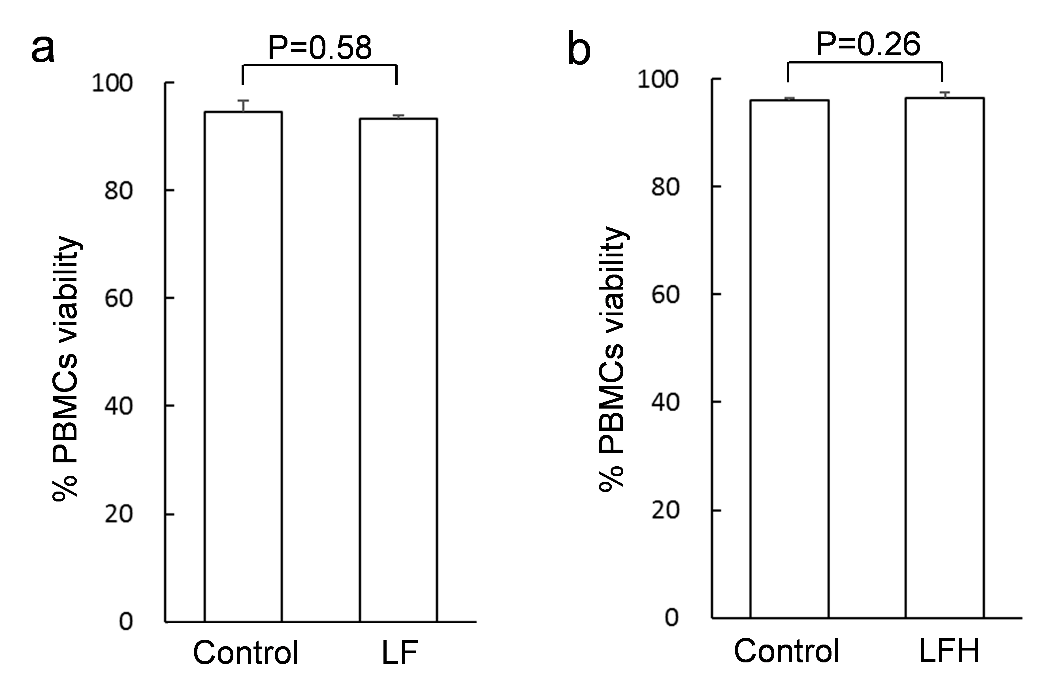


**Supplementary Fig. S2** Viability of peripheral blood mononuclear cells (PBMCs) treated with water (control) and 100 µg/mL of lactoferrin (LF) (a) or its pepsin hydrolysate (LFH) (b) for 24 h. The bars and error bars represent the mean and standard deviation (n = 3).


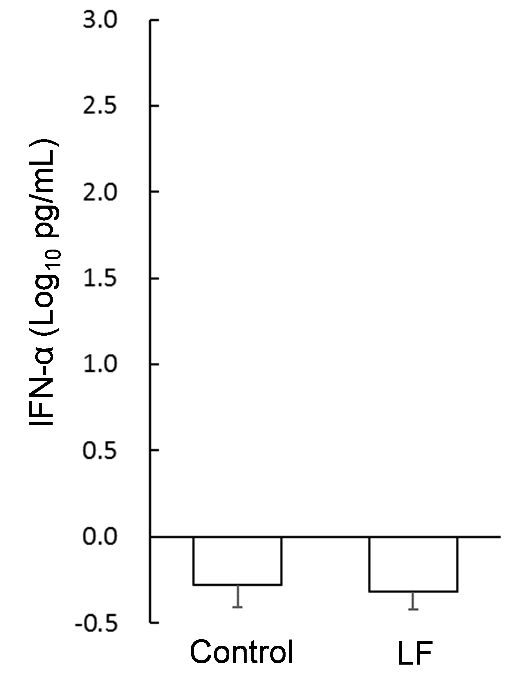


**Supplementary Fig. S3** Concentrations of interferon (IFN)-α in the culture supernatants of PBMCs treated with water (control) or 100 µg/mL of LF in the absence of single-stranded RNA (ssRNA) for 24 h. The bars and error bars represent the mean and standard deviation (n = 4).


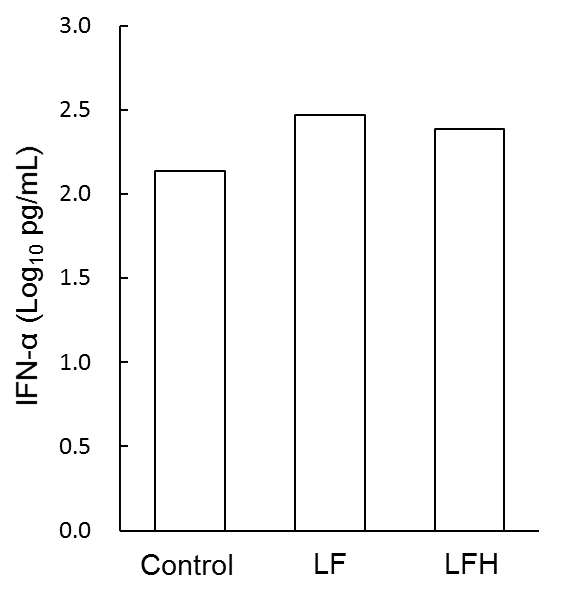


**Supplementary Fig. S4** Concentrations of IFN-α in the culture supernatants of PBMCs from one donor treated with water (control), 100 µg/mL of LF or 100 µg/mL of LFH in the presence of single-stranded RNA (ssRNA) for 24 h. (n=1)


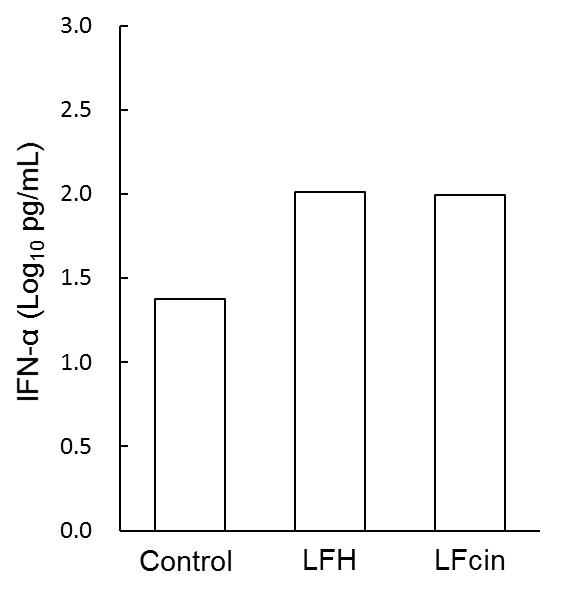


**Supplementary Fig. S5** Concentrations of IFN-α in the culture supernatants of PBMCs from one donor treated with water (control), 100 µg/mL of LFH or 4 µg/mL of LFcin in the presence of single-stranded RNA (ssRNA) for 24 h. (n=1)


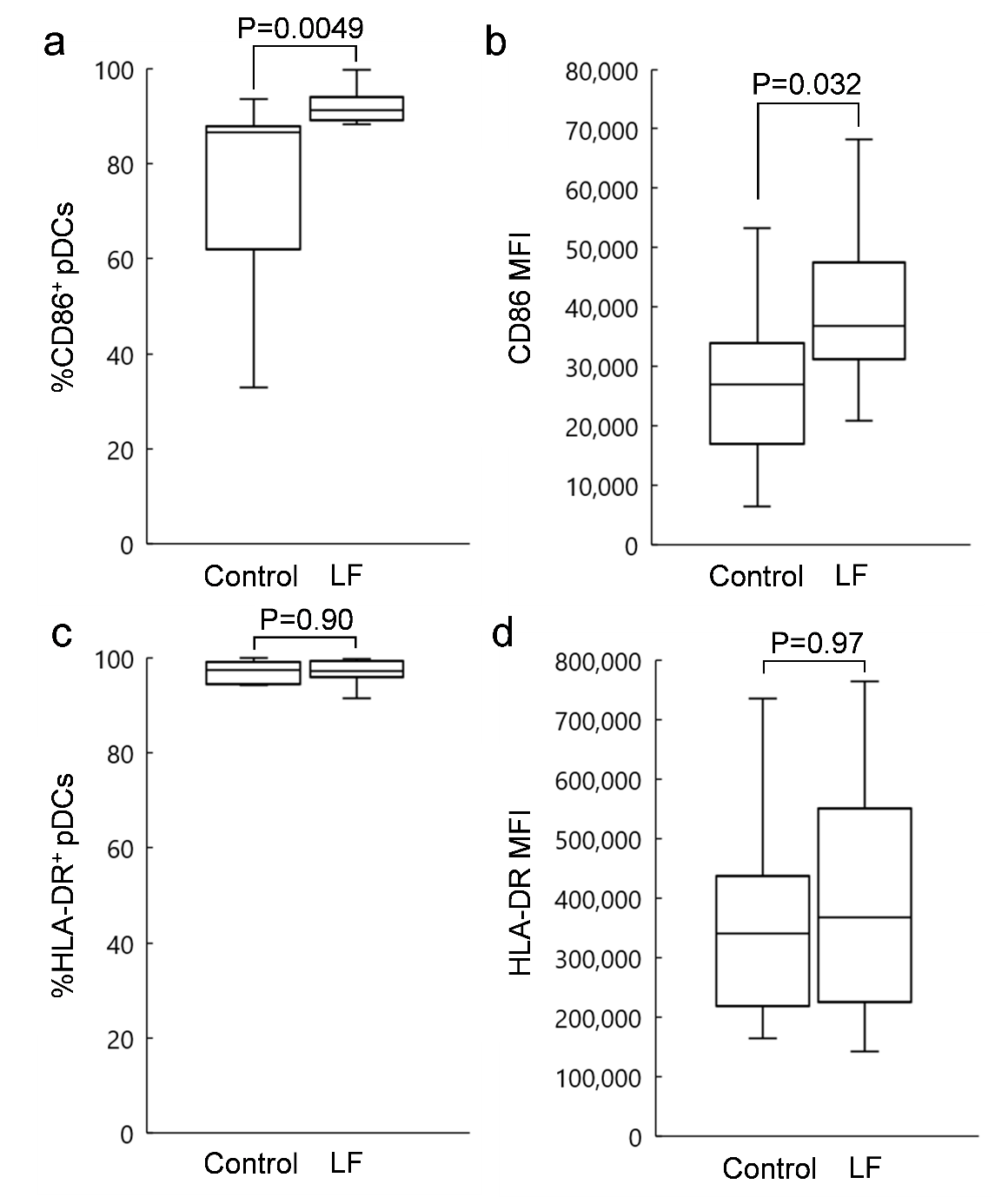


**Supplementary Fig. S6** Percentage of CD86^+^ plasmacytoid dendritic cells (pDCs) in total pDCs (a), median fluorescence intensity (MFI) of CD86 in pDCs (b), percentage of human leukocyte antigen (HLA)-DR^+^ pDCs in total pDCs (c), and MFI of HLA-DR in pDCs (d) treated with water (control) or 100 µg/mL of LF in the absence of ssRNA for 24 h. The horizontal line indicates the median, the box covers the 25–75^th^ percentiles, and the vertical whiskers show the highest and lowest values excluding outliers

(n = 11). Graphs were reconstituted from data of our previous report (Kubo et al. 2021).
